# Strategies to improve participation in exercise programmes during chemotherapy: a modified nominal group technique

**DOI:** 10.1007/s11764-025-01771-y

**Published:** 2025-03-07

**Authors:** N. Kearney, D. Connolly, K. Bahramian, E. Guinan

**Affiliations:** 1 Department of Surgery, School of Medicine, Trinity College Dublin, Dublin, Ireland; 2 Trinity St James’S Cancer Institute, St James’S Hospital, Dublin 8, Ireland; 3 Discipline of Occupational Therapy, School of Medicine, Trinity College Dublin, Dublin, Ireland; 4 OvaCare, Cork, Ireland; 5 Discipline of Physiotherapy, School of Medicine, Trinity College Dublin, Dublin, Ireland

**Keywords:** Exercise, Chemotherapy, Recruitment, Adherence, Retention

## Abstract

**Purpose:**

Exercise can help people manage many of the side effects of chemotherapy treatment. Clear guidelines exist outlining the benefits of exercise during chemotherapy and recommended dosage; however, achieving these guidelines remains problematic. The purpose of this study is to reach an agreement on suitable strategies to improve recruitment, adherence, and retention rates to exercise programmes during chemotherapy with the involvement of key stakeholders.

**Methods:**

This study used a modified nominal group technique (NGT). Participants included individuals with a lived experience of chemotherapy and healthcare professionals working in oncology. Three workshops were carried out, two in-person and one online. The in-person workshops addressed the first four stages of the NGT, introduction, idea generation, round-robin recording, and group discussion. Voting and ranking of ideas occurred during the online workshop.

**Results:**

Nineteen individuals took part in this study, including 12 people with a lived experience of chemotherapy and seven healthcare professionals. The highest-ranked strategy to improve recruitment was to inform individuals about the benefits of exercise at the time of receiving their treatment plan, with 53% of first preference votes. Participants also agreed that direct instruction from their oncologist would have the greatest impact on successful recruitment to an exercise programme, receiving 47% of first preference votes. To enhance exercise adherence, an in-person exercise programme delivered in an exercise facility received 46% of first preference votes. Finally, 43% of participants agreed that the provision of a pedometer would support retention, and 86% of people wanted to receive weekly phone calls/check-ins.

**Conclusion:**

This study provides strategies to overcome barriers to recruitment, adherence, and retention to exercise programmes during chemotherapy, and will help to optimise participant engagement for future interventions.

**Implications for Cancer Survivors:**

The involvement of key stakeholders in this study will contribute towards ensuring that future interventions are pragmatic and patient-centred.

## Introduction

Overall cancer mortality rates have reduced by approximately 15% over the last 30 years [1]. While the development of new chemotherapy regimens is having a significant impact on reducing mortality rates, concerns around the side effects and toxicities of these drugs still remain. Anthracycline-based chemotherapies for example are strongly linked with an increased risk of cardiotoxicities [2], taxanes may lead to neurotoxicities [3], and haematological toxicities such as neutropenia and anaemia are also common [4]. These toxicities can be dose-limiting as they can often result in dose reductions or dose delays which can compromise treatment efficacy and may lead to poorer prognosis [5, 6]. In addition to this, toxicities can give rise to multiple side effects including fatigue, nausea, muscle weakness, neuropathy, and pain, which can negatively impact patients’ physical function and quality of life during and after treatment [7, 8]. Strategies to alleviate the impact of these dose-limiting toxicities (DLTs) are therefore critical to improve chemotherapy tolerance and optimise efficacy.

There is now substantial evidence supporting the benefits of exercise for managing many of the toxicities and side effects associated with chemotherapy, and thus, improving people’s quality of life during this time [9, 10]. Such is the importance of exercise; The American Society of Clinical Oncology (ASCO) provides clear instructions for oncology providers to recommend regular aerobic and resistance exercise during active treatment to mitigate many side effects of chemotherapy treatment [11]. The exercise guidelines for individuals during chemotherapy have been outlined by The American College of Sports Medicine (ACSM) which recommends moderate-intensity aerobic exercise 3 days per week, for at least 30 min per day. In addition to this, it is also recommended to engage in resistance training 2 days per week [12]. The exercise guidelines for people during chemotherapy are clearly stated, but achieving these guidelines remains problematic.

Individuals are faced with a number of physical and logistical barriers to exercise participation during chemotherapy, and therefore it is unsurprising that many people fail to meet the recommended exercise guidelines during this time [13]. A recent cross-sectional study examining barriers and facilitators to exercise among adult cancer survivors during chemotherapy reported that adverse effects from treatment (46.2%), lack of self-discipline (29.2%), and lack of time (26.2%) were the biggest barriers to exercise participation during this time [14]. Healthcare professionals also report barriers to implementing services during chemotherapy. A cross-sectional study involving physicians and oncology nurses reported not having enough time with patients, lack of available programmes, and lack of an expert contact person as the primary barriers to promoting exercise to their patients [15].

We recently systematically reviewed key feasibility metrics of exercise trials during chemotherapy in order to quantify recruitment, adherence, and retention rates in these trials [16]. Mean recruitment rates for these trials were 62%, with travel constraints (18.7%), lack of interest (15.4%), and lack of time (11.6%) cited as the main barriers to recruitment. Programme adherence was suboptimal, particularly for resistance exercise (67.6%), and poorly quantified across trials. Mean retention rates were 84.1%, while the main reasons for dropping out of these trials were due to chemotherapy side effects (15%), unwillingness to exercise (11.1%), and being too busy (8.1%). Results from this review also showed that there is a lack of diversity in the demographics of people who participate in these studies. This review highlighted the multiple challenges that exist when implementing exercise trials during chemotherapy, and that identifying strategies to optimise recruitment, adherence, and retention to programmes during this time is imperative.

Recognising the ever-growing need for patient-centred care, co-design frameworks have attracted a lot of attention in healthcare research in recent years [17]. Co-design involves collaboration with key stakeholders such as people with lived experience and healthcare professionals to help design products and services [18]. Co-design frameworks are listed second on the Ladder of Co-production which indicates that research is conducted with service users rather than for them [18]. This collaborative co-design approach helps to ensure that interventions are not only evidence-based but also tailored to the individuals for whom they are targeted. Co-design frameworks have not been widely used in exercise oncology trials to date [19–23] which suggests that the majority of interventions that are being developed do not consider the individual’s needs, and therefore lack relevance to the service users. Gathering input from user groups in the design process is important to ensure that interventions that are developed are pragmatic and user-friendly. The primary aim of this study was to reach an agreement on suitable strategies to increase rates of recruitment, adherence, and retention to exercise programmes during chemotherapy with the involvement of key stakeholders.

## Methods

### Study design

**Fig. 1:**
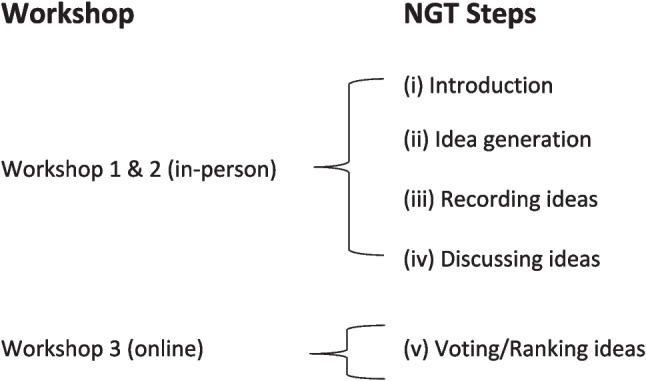
Study design

This study used a modified nominal group technique (NGT) to collect both quantitative and qualitative data through a series of co-design workshops. The NGT is a collaborative brainstorming approach used to reach consensus through the following five steps: (i) introduction, (ii) idea generation, (iii) recording ideas, (iv) discussing/clarifying ideas, and (v) ranking ideas. Traditionally, these five steps are carried out during a single in-person workshop; however, modified approaches which complete these steps across different workshops have been described [24]. The modifications of the NGT that were applied in this study included completing the NGT over three individual workshops using a hybrid delivery model, including two in-person workshops and one virtual workshop. The two in-person workshops addressed the first four steps of the NGT which focused on introduction, idea generation, recording of ideas, and group discussion. Both in-person workshops followed the same format but were held in different locations, with different participants, to facilitate the inclusion of rural and urban participants. All participants who attended the in-person workshops were then invited to attend the online workshop which involved the final step of the NGT, voting and ranking of ideas (Fig. 1). This modified approach allowed the researchers time to combine all ideas generated from the first two workshops and to develop statements for voting for the virtual workshop.

Ethical approval was granted for this study in May 2023 from the Faculty of Health Sciences Research Ethics Committee, Trinity College Dublin [REF: 230410].

### Participants

Workshops were attended by people with a lived experience of chemotherapy and healthcare professionals (HCPs). People with lived experience included anyone who had previously received or was currently receiving chemotherapy for cancer, family members, or caregivers. These individuals were recruited from local community cancer support centres, the Irish Cancer Society, as well as some established public and patient involvement (PPI) network groups. Healthcare professionals included physiotherapists, nurses, occupational therapists, or any other relevant disciplines working within the area of oncology. Healthcare professionals were recruited through local hospitals, community healthcare organisations, and staff working in cancer support centres.

Recommendations from previous research suggested that each NGT group should include between five to nine participants [25], while other studies recommended no more than seven participants per group [26]. For this reason, participants in each NGT were sub-divided into groups of five to six members with an equal mix of people with a lived experience of chemotherapy and HCPs. Each sub-group was facilitated by a member of the research team (DC, KB, and EG).

### PPI governance and leadership

All study activities were overseen by a core PPI partner (KB) who formed part of the study steering committee. This PPI partner played a key role in planning, facilitating, and debriefing following the codesign workshops. Having a PPI partner as part of the steering committee ensured that the study procedures were carried out as planned from the outset. In addition to this, the PPI partner also helped to communicate the study in lay terms to participants and played a role in group facilitation.

### Study procedures

#### NGT steps 1 to 4: idea generation

Two in-person workshops were held as part of this study. The first in-person workshop took place in a rural location in Tullamore Library, Co. Offaly, while the second workshop was held in an urban setting in the Trinity Centre for Health Sciences, St James's Hospital, Dublin. Both workshops followed the same format and participants attended the workshop that was most convenient for them.

During the in-person workshops, participants were asked to consider strategies to improve rates of recruitment, exercise adherence, and retention to an exercise programme during chemotherapy. These three elements are key feasibility metrics when designing exercise interventions, as identified from a systematic review carried out by the research team [16]. The specific questions that were posed to the participants during the in-person workshops included:

1. What strategies would optimise recruitment to an exercise programme delivered during chemotherapy?
2. What strategies would facilitate adherence to an exercise programme during chemotherapy?
3. What strategies would support the retention of an exercise programme during chemotherapy?

The workshop facilitator (NK) presented and explained each question in lay terms and then participants were given 30–40 min to silently generate as many strategies for each question as possible. Participants recorded their ideas on Post-it notes which were colour-coded to match each question. A facilitator (KB, DC, and EG) was present at each sub-group to limit discussion during the silent generation of ideas and answer questions as necessary. As participants were generating their ideas, the workshop facilitator (NK) collected the ideas and recorded them on a flipchart. Following the completion of the silent generation of ideas for all questions, a facilitated group discussion took place to clarify and refine the strategies generated.

#### NGT step 5: voting and ranking

The online workshop took place 3 weeks after the two in-person workshops and was carried out via Zoom Video Communications. This online workshop completed the final step of the NGT, voting and ranking of ideas. All participants who attended the in-person workshops were invited to attend.

In this workshop, participants were provided with a list of strategies that were generated during the in-person workshops and were asked to rank each strategy in order of preference. In the NGT, the number of ideas generated can vary depending on the question; however, rankings of five ideas are most common in the literature [24]. Higher idea ranking represents higher preference (e.g., 5 = highest preference, 1 = lowest preference). The total number of votes for each question was tallied up to determine the highest-ranked strategies.

### Data analysis

Ideas generated during the two in-person workshops were categorised using summative content analysis [27]. This approach involved quantifying the presence of recurring keywords during the idea generation to identify the most common ideas and strategies. This process allowed the research team to group identified strategies in preparation for voting during the online workshop. Summative content analysis is often used to analyse large amounts of textual data and has been recommended for use in group-working and consensus-building activities [28].

Voting during the online workshop was performed using the Mentimeter platform which captured results in real time. Results were automatically populated into an Excel spreadsheet following the workshop highlighting the individual voting preferences for each question.

## Results

### Participant characteristics

A total of 19 participants agreed to participate in this study. Eight participants attended the first in-person workshop and 11 attended the second in-person workshop. Participants in the two workshops included 12 people with a lived experience of chemotherapy and seven healthcare professionals working in oncology services. Of the people with a lived experience of chemotherapy, eight had previously received chemotherapy, three were currently undergoing treatment, and one was a carer for a family member who had received chemotherapy. Six participants had a history of breast cancer diagnosis, and five participants had a history of gynaecological cancer. All participants were female with a mean age of 56 years (SD = 8.0; range 43–66). Healthcare professionals included nurses (*n* = 4), physiotherapists (*n* = 2), and a community cancer support centre manager (*n* = 1). Healthcare professionals’ years of experience working with people during chemotherapy ranged from 1 to 30 years (median = 10 years).

Fifteen participants returned for the voting workshop for the ranking of strategies. Eleven were people with a lived experience of chemotherapy, and four were healthcare professionals.

### Idea generation (workshops 1 and 2)

Results from the idea generation phase identified a number of sub-categories for each of the questions posed (Table 1). These sub-categories formed the statements for voting in the online workshop.

**Table 1:** Results from idea generation workshops

	Sub-categories identified during idea generation	Recommendations for voting
Recruitment	**Optimal timing of recruitment**	• At the very beginning, when the oncologist is discussing the treatment plan • On the day of the first chemotherapy infusion • Between the first and second chemotherapy cycle • Between the second and third chemotherapy cycle
	**Optimal method of recruitment**	• Direct advice from a member of the oncology team • An incentive such as a FitBit, pedometer, exercise calendar • Simple information packs to take home • Testimonials from peers
Adherence	**Optimal method of exercise delivery**	• Group exercise classes in an exercise facility with peers going through similar treatment • Group exercise classes online via Zoom with peers going through similar treatment • Exercise alone using exercise videos/tutorials • A combination of all of the above
Retention	**Frequency of monitoring**	• Once per week • Twice per week
	**Optimal motivational strategies**	• An event/goal to aim for • FitBit/pedometer to reach weekly step goals • Parking ticket/expenses • Exercise calendar to mark off the days exercised • Badge/certificates for achieving milestones

In response to the first question on strategies to optimise recruitment, two sub-categories were identified: (1) the optimal timing of recruitment and (2) the optimal methods of recruitment. When exploring strategies to optimise exercise adherence, the majority of recommendations are related to the optimal method of exercise delivery. Lastly, when asked about strategies to optimise retention, the key sub-categories that arose during the idea generation included (1) the frequency of monitoring/check-ins and (2) optimal motivation strategies. These two sub-categories formed the final two questions for voting during the online workshop.

The sub-categories which were generated during the idea generation phase and the recommended strategies are highlighted in Table 1.

### Recurring ideas

Some recurring keywords were identified from the idea generation that were consistent throughout both in-person workshops. Keywords during the content analysis helped to identify unanimous ideas that participants agreed should be part of any exercise programme delivered during chemotherapy. The recurring keywords during idea generation included; “fun”, “individualised”, “peer support”, “education”, “flexibility”, “motivation”, and “autonomy”.

### Consensus/voting (workshop 3)

#### Strategies to improve recruitment

##### Timing of recruitment

During the online workshop, participants were presented with options relating to the most appropriate time to receive information about exercise during chemotherapy. The highest ranked preference for the optimal timing of recruitment to an exercise programme was at the first meeting when the oncologist was discussing the individual’s chemotherapy plan, receiving a total rank score of 41/60, and eight participants (53%) gave this their first preference vote. The second most preferred strategy was to recruit participants between their first and second chemotherapy cycles (Table 2)

**Table 2:** Ranked preferences for strategies to support recruitment

Category for voting	Ideas	Individual rank scores	Total ranks scores	Number of votes
(i) Optimal timing of recruitment	At the very beginning, when the oncologist is discussing the treatment plan	4, 4, 4, 4, 4, 4, 4, 4, 3, 1, 1, 1, 1, 1, 1	41	15
	Between the first and second chemotherapy cycle	4, 3, 3, 3, 3, 3, 3, 3, 3, 2, 2, 2, 2, 2, 1	39	15
	On the day of the first chemotherapy infusion	4, 4, 4, 4, 3, 3, 3, 3, 2, 2, 2, 1, 1, 1	37	14*
	Between the second and third chemotherapy cycle	4, 4, 3, 3, 2, 2, 2, 2, 2, 2, 2, 1, 1, 1, 1	32	15
(ii) Optimal method of recruitment	Direct advice from a member of the oncology team	4, 4, 4, 4, 4, 4, 4, 3, 3, 3, 3, 2, 2, 2, 1	47	15
	Testimonials from peers	4, 4, 4, 4, 4, 3, 3, 3, 3, 2, 2, 2, 1, 1, 1	41	15
	Simple information packs to take home	4, 4, 3, 3, 3, 3, 2, 2, 2, 1, 1, 1, 1, 1, 1	32	15
	An incentive such as FitBit, pedometer, exercise calendar	4, 3, 3, 3, 2, 2, 2, 2, 2, 2, 1, 1, 1, 1, 1	30	15

^*^One participant failed to record their vote due to technical difficulties

##### Method of recruitment

To reach a consensus on the sub-category of the optimal method of recruitment, participants voted on their preferred strategies relating to “what would have the biggest influence on people’s decision to start an exercise programme during chemotherapy”. In response to this, the consensus was that direct advice from a member of the oncology team would have the biggest impact when recruiting people to an exercise programme. This strategy received a total rank score of 47/60 with seven participants (47%) giving this their first preference vote (Table 2).

#### Strategies to improve exercise adherence

During the online workshop, participants were asked to rank their preferred method of exercise delivery to support exercise adherence. The strategy which received the most votes was ‘group-based exercise classes in an exercise facility with peers going through similar treatment’ receiving a total rank score of 39/52 and 46% of first preference votes. The least preferred method of exercise delivery was to exercise alone using exercise videos or tutorials (Table 3).

**Table 3:** Ranked preferences for strategies to support exercise adherence

Ideas	Individual rank scores	Total rank scores	Number of votes
Group exercise classes in an exercise facility with peers going through similar treatment	4, 4, 4, 4, 4, 4, 3, 3, 3, 2, 2, 1, 1	39	13*
Group exercise classes online via Zoom with peers going through similar treatment	4, 4, 3, 3, 3, 3, 3, 2, 2, 1, 1, 1, 1	31	13*
Exercise alone using exercise videos/tutorials	4, 3, 3, 3, 3, 2, 2, 2, 2, 2, 1, 1, 1	29	13*
A combination of all of the above	4, 4, 4, 4, 4, 3, 2, 2, 2, 2, 1, 1, 1, 1	33	14*

^*^Some participants failed to record their votes due to technical difficulties

#### Strategies to improve retention

##### Monitoring/check-ins

During the online workshop, participants voted on the optimal frequency of checking in and monitoring an exercise programme to support retention. The consensus showed that participants would prefer weekly check-ins as opposed to biweekly, receiving a total rank score of 26/28 and 12 out of 14 first-preference votes (86%). (Table 4).

**Table 4:** Ranked preferences for strategies to support retention

Category for voting	Ideas	Individual rank scores	Total ranks scores	Number of votes
(i) Frequency of monitoring	Once per week	2, 2, 2, 2, 2, 2, 2, 2, 2, 2, 2, 2, 1, 1	26	14*
	Twice per week	2, 2, 1, 1, 1, 1, 1, 1, 1, 1, 1, 1, 1	15	13*
(ii) Optimal motivational strategies	FitBit/pedometer to reach weekly step goals	5, 5, 5, 5, 5, 5, 4, 4, 4, 3, 4, 4, 4, 4	53	14*
	An event/goal to aim for	5, 5, 5, 5, 5, 4, 4, 4, 4, 3, 3, 2, 2, 2	53	14*
	Badges/certificates for achieving milestones	5, 4, 4, 4, 3, 3, 3, 3, 3, 3, 2, 2, 2, 1	42	14*
	Exercise calendar to mark off the days you exercised	5, 5, 4, 4, 4, 4, 3, 3, 2, 1, 1, 1, 1, 1	39	14*

^*^Some participants failed to record their votes due to technical difficulties

##### Motivation strategies

When voting on preferred motivational strategies to support retention to an exercise programme over time, results showed that participants ranked an incentive such as a Fitbit/pedometer, and an event/goal to aim for, as joint first preference with both receiving a total rank score of 53/70, although a Fitbit/pedometer received more first preference votes (43%) compared to an event/goal to aim for (36%). Table 4 shows a detailed breakdown of participants’ preferences towards staying motivated to exercise over time.

## Discussion

This study was conducted to seek consensus from different stakeholder groups on strategies to enhance recruitment, exercise adherence, and retention to an exercise programme during chemotherapy. The NGT study design facilitated people with a lived experience of chemotherapy and oncology healthcare professionals to voice their opinions on optimal strategies to consider when delivering an exercise programme during chemotherapy. The initial phases of this study identified a range of strategies which could help overcome many of the identified barriers to exercise participation during chemotherapy. The nominal group technique provided a robust methodology for reaching consensus in this study.

Participants in the current study agreed that the benefits of participating in an exercise programme should be discussed with them as part of their overall treatment plan, at the time the oncologist is discussing their treatment plan. This has also been echoed in a previous information needs assessment where patients reported that they would prefer to receive information on exercise prior to beginning treatment [29]. These preferences are supported by the results of a recent systematic review where it was reported that recruitment rates were higher for exercise programmes that recruited participants before they began their chemotherapy treatment (70.5%) compared to programmes recruiting people after beginning treatment (57.6%) [16]. It is important to note that starting an exercise programme before beginning chemotherapy may not be feasible for all patients [14]. It is reported that approximately 75% of cancer patients report being overwhelmed by the information they receive after their cancer diagnosis—a concept known as cancer information overload (CIO) [30]. Findings from this NGT suggest that recruitment to an exercise programme should be attempted before beginning chemotherapy treatment; however, recruiting people after the first or second chemotherapy cycle may be a viable alternative if patients feel overwhelmed at the time of receiving their treatment plan.

Participants in this study also agreed that direct instruction from their nurse or oncologist would help to support recruitment to an exercise programme during chemotherapy. In a cross-sectional study examining exercise discussions during cancer treatment, 82.2% of patients believed that the oncologist should initiate a discussion around exercise during their chemotherapy consultation [31]. In addition to this, another study reported that 57% of participants preferred to receive recommendations about exercise from their oncologist, while only 30% wanted to receive this information from a physiotherapist [32]. There is clear evidence showing that direct instruction from an oncologist is associated with greater rates of recruitment and exercise participation in exercise oncology trials [31, 33–35]. Despite the important role of the oncologist in the recruitment process, studies have shown that as little as 19–24% [31, 36] of patients receive information from their oncologist about exercising during treatment. New guidelines from the American Society of Clinical Oncology (ASCO) which calls for “oncology providers to recommend regular aerobic and resistance exercise during active treatment” [11] highlight the need to embed exercise into the overall cancer care pathway and encourage oncologists to include it as part of their overall treatment plan. Frameworks have been put in place to aid oncology providers in recommending exercise to their patients such as the “Exercise is Medicine” initiative which encourages oncologists to “Assess, Advise, and Refer” their patients to exercise programmes during treatment [37]. Similarly, the Making Every Contact Count (MECC) initiative encourages healthcare professionals to support their patients to make healthier lifestyle choices which include promoting regular physical activity and exercise [38].

One of the main barriers to oncologist-led recruitment, however, is that oncologists report having a lack of time with patients or that they see exercise as a low priority, making the applicability of oncologist referral difficult in practice [39]. To overcome these barriers, nurses can play an important role in this discussion due to more frequent interactions and more time spent with patients. Despite this, it has been found that only 9% of oncology nurses talk to all of their patients about physical activity [40], and 67% report having insufficient knowledge regarding exercise in cancer care [41]. With sufficient education and training, nurses have a unique opportunity to promote the benefits of exercise to patients during chemotherapy and may play a significant role in aiding recruitment in exercise oncology trials [42].

Participants in this current study agreed that they would most likely adhere to an exercise programme if they attended group exercise classes in an exercise facility with peers receiving similar treatment. These preferences are in line with those reported in a recent cross-sectional study which reported that the majority of cancer patients wanted to exercise in a group with other cancer patients [32]. Peer support is recognised to promote mental well-being and also increases patient’s perceived benefit of exercise which may help to optimise adherence [43]. Considering that travel was identified as one of the greatest barriers to exercise participation in exercise oncology trials [16], there is a great need for more exercise facilities in the community to overcome this barrier and facilitate this preference for in-person exercise. Delivery of in-person exercise may be more beneficial for exercise selection, technique, safety, prescribing intensity, and accountability and may also provide a greater social aspect compared to home-based programmes, all of which may contribute towards achieving higher adherence rates [44]. Although there is inconclusive evidence directly linking in-person exercise to better adherence rates [16, 45], findings from this current study suggest that it is still important that a programme offers opportunities for in-person exercise classes during chemotherapy.

Regular monitoring and consultations with an exercise professional are an integral part of any exercise programme. Telephone contact gives exercise professionals an opportunity to discuss aspects of their training programme with patients, modify exercises, answer any questions, and set goals for the coming weeks [46, 47]. Qualitative feedback shows that weekly telephone calls also give patients a sense of care, and comfort and increase their accountability which could be contributing factors to improving adherence and retention to exercise programmes during chemotherapy [48, 49]. Multiple studies have shown the beneficial effects of weekly check-ins, including one study which successfully delivered a telephone-based-only exercise intervention for cancer survivors [50]. In addition to this, Borsati et al. [47] showed that weekly monitoring through telephone calls was a strong stimulus to prevent dropout in an exercise programme in cancer survivors. Although regular contact with patients can be beneficial for improving retention, it is also important that check-ins are not overly burdensome. Some patients may have a feeling of guilt if they are unable to meet their desired exercise targets, and constant monitoring might have the opposite effect on retention as patients may feel under pressure and become discouraged [51]. Results from this NGT indicate that participants believe that a weekly check-in phone call is sufficient without being too burdensome.

Participants in this NGT also agreed that an incentive such as a Fitbit/pedometer, and an event/goal to aim for are suitable strategies to support retention to an exercise programme during chemotherapy. Pedometers and other wearable activity trackers have been shown to be effective tools to help increase physical activity levels over time [52]. Findings from a systematic review suggested that pedometers can be used as an effective motivational tool when coupled with an exercise goal such as a specified number of daily steps [53]. Widely available smartphone trackers produce comparable results to more expensive pedometer trackers which supports their use for promoting long-term physical activity in cancer patients due to ease of accessibility [54]. In addition to this, goal-setting is widely used in exercise oncology resulting in positive findings on improving exercise adherence and retention rates [55]. Individual goal-setting is recognised as a vital component to ensure exercise adherence in clinical practice [56]. Embedding individual goal-setting and incentives such as pedometers or activity trackers show great potential to keep patients motivated and engaged and may aid with long-term retention rates.

Numerous recommendations were identified from the group discussions during the in-person workshops which participants agreed should be a part of any exercise programme targeted for patients undergoing chemotherapy. Some of these recommendations included that the exercise be flexible, individualised, fun, and broken down into small ‘exercise snacks’. Participants in this current study agreed that exercise should be individualised not only based on age, gender, and exercise history but also individualised based on daily symptoms and energy levels. Autoregulation is an exercise training concept that allows individuals to self-adjust their training variables based on their own subjective feelings of fatigue and their daily readiness to exercise [57]. Using the BORG rating of perceived exertion (RPE) scale [58] is an effective way to allow patients to autoregulate exercise at their own individualised intensity based on motivation and ability on a given day while also supporting self-management. There has also been growing evidence over the last number of years supporting the incorporation of ‘exercise snacks’ to improve the health outcomes of individuals living with cancer [59]. Exercise snacks involve short bouts of exercise dispersed throughout the day and have shown promise for improving cancer-related fatigue, and cardiometabolic health and may also improve cardiorespiratory fitness [59, 60]. Participants in the NGT agreed that small bouts of exercise throughout the day would be more attainable than continuous, uninterrupted exercise sessions.

## Strengths and limitations

One of the strengths of this study was the inclusion of a wide range of key stakeholders including people with a lived experience of cancer, family members, and healthcare professionals from different disciplines. This encouraged comprehensive idea generation to each question from people with different backgrounds. In addition to this, the NGT approach encouraged equal contribution from all participants and eliminated the possibility of some members dominating the discussions. Another strength of this study was that two in-person workshops were held in different geographical locations to facilitate inclusion for people living in urban and rural areas.

A limitation of this study was the lack of diversity in the participants who took part in this study. Firstly, all participants in this study were female. Despite our best efforts, we were unable to recruit any males to participate in this study which is a limiting factor. In addition to this, all participants involved in the workshops had a keen interest in exercise. We were unable to recruit individuals with no personal interest in exercise. There was also a lack of diversity in the cancer types of those involved in this study, with only two different cancer types being represented in these workshops. It would be useful to include participants with all different cancer types as people with different cancers receive different levels of support and will have different barriers to exercise participation. The authors also had no information on the socioeconomic status of the participants involved in this study. It is possible that people from different socioeconomic backgrounds would have different preferences to meet their individual needs.

## Conclusion

It is well understood that multiple barriers exist when recruiting and retaining individuals to an exercise programme during chemotherapy. Exploring strategies to optimise rates of recruitment, adherence, and retention to exercise programmes during chemotherapy is imperative to improve the feasibility of how these programmes are being delivered. With a growing recognition for patient-centred care, this is the first study to involve key stakeholders in designing an exercise programme for patients during chemotherapy. Involving people with a lived experience of cancer and oncology healthcare professionals in the design process will help to overcome many of the barriers to exercise participation during chemotherapy and ensure that future programmes are pragmatic and patient-centred.

## Data Availability

The raw data generated during and analysed during the current study are available from the corresponding author on reasonable request.
